# Systematic Review and Meta-analysis of Alarm versus Desmopressin Therapy for Pediatric Monosymptomatic Enuresis

**DOI:** 10.1038/s41598-018-34935-1

**Published:** 2018-11-13

**Authors:** Carol Chiung-Hui Peng, Stephen Shei-Dei Yang, Paul F. Austin, Shang-Jen Chang

**Affiliations:** 1 Department of Internal Medicine, University of Maryland Medical Center Midtown Campus, Baltimore, Maryland 21201 USA; 2 Division of Urology, Taipei Tzu Chi Hospital, Buddhist Tzu Chi Medical Foundation, New Taipei City, 231 Taiwan; 3 School of Medicine, Buddhist Tzu Chi University, Hualien, 970 Taiwan; 4 Department of Urology, Texas Children’s Hospital and Baylor College of Medicine, Houston, Texas 77030 USA

## Abstract

This study is to compare the efficacy of enuresis alarm and desmopressin therapy in managing pediatric monosymptomatic enuresis. We performed systematic literature searches on different databases from inception until April 2017 without language restriction. All randomized control trials comparing an enuresis alarm and desmopressin in managing children with monosymptomatic enuresis were included. A total of 15 studies with 1502 participants (aged 5 to 16 years) were included for pooled analysis. Overall, an enuresis alarm outperformed desmopressin in achieving at least a partial response (>50% reduction in wet nights) in per-protocol analysis (OR: 1.53, 95% CI 1.05 to 2.23) but not in intention-to-treat analysis (OR: 0.97, 95% CI 0.73 to 1.30) as the alarm was hampered by a high dropout rate (OR: 2.20, 95% CI 3.41 to 4.29). However, alarm therapy yielded a better sustained response (OR: 2.89, 95% CI 1.38 to 6.04) and lower relapse rate (OR: 0.25, 95% CI 0.12 to 0.50). In the intention to treat analysis, the results revealed that alarm and desmopressin therapy are comparable in efficacy with regards to achieving >50% reduction in baseline wet nights in enuretic children. However, enuresis alarms offer a superior treatment response and a lower relapse rate in well-motivated children.

## Introduction

Nocturnal enuresis is common in children, especially boys, with a prevalence ranging from 5–20% at 5 years of age depending on area, race and definition used^1–5^. Active treatment is warranted not until 5 years of age owing to its self-limiting feature^6^. Enuresis appears to affect self-esteem in patients despite its frequency. Treatment with various modalities showed to improve self-worth on children, regardless of outcome^7^. The International Children’s Continence Society (ICCS’) recommend alarm therapy should be considered in every child with NE, but especially in those with well-motivated parents^6^. In addition to alarm therapy, desmopressin is also regarded as a first line therapy with grade Ia evidence.

Enuresis alarms have been used to treat pediatric nocturnal enuresis for decades. These alarms work as moisture sensors and are attached to underwear or bed pads and give audio or vibrating signals. Among different designs, the body sensor yielded better outcome when compared with the pad sensor^8^. Alarm therapy warranted cooperative parents and children with proper training programs to improve compliance and success rate. Alarm therapy results in dryness in about two thirds of children. The manner in which the conditioning treatment is presented and monitored affects the success rate^9^. Many types of alarms, variable protocols and additional behavioral interventions (i.e. star charts, reward systems, overlearning, retention control training, urine stream interruption exercises, lifting and scheduled wakening) have been proposed to improve success rate and lower relapse rate^10^. The most impressive outcome data were published by Foxx and Azrin’s Dry Bed Training and Houts’ Overlearning^11,12^.

Desmopressin, a synthetic vasopressin analogue, has been used in clinical practice to manage nocturnal enuresis for decades with promising efficacy^13^. Desmopressin could be given with intranasal, oral tablets and oral melt lyophilisate form^14^. However, the indication of intranasal route desmopressin for pedicatric nocturnal enuresis was withdrawn by FDA due to possible hyponatremia, seizures and death. The ordinary dose is 0.2 to 0.4 mg and 120 to 240 μg for the oral tablet and oral melt forms, respectively^15^. Variable dosing strategies and withdrawal methods have been reported to improve outcomes.

At present, no detailed systematic reviews or meta-analyses have compared the two first-line treatment modalities. Also, most randomized control trials comparing the two management strategies have included relatively small sample sizes with significant variations in participant characteristics and outcome measurements, they lack statistical power to assess differences between the two therapies. Therefore, a systemic review and pooled analysis may help pediatric urologists to more clearly clarify the differences. The aim of this systemic review and meta-analysis was to compare the initial treatment response, relapse rate and sustained response rate between an enuresis alarm and desmopressin therapy in children with monosymptomatic nocturnal enuresis.

## Results

### Study selection

**Figure 1:**
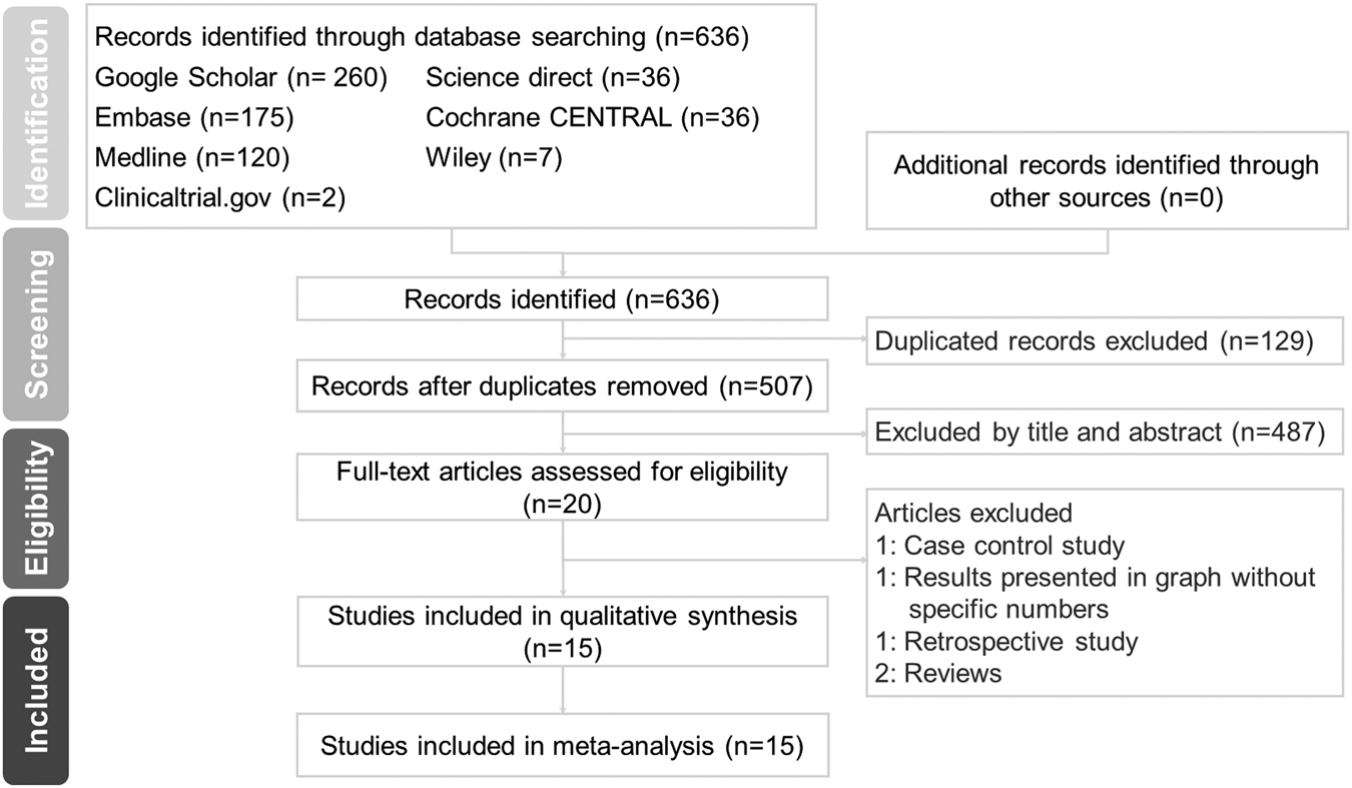
Preferred reporting items for systematic reviews and meta-analyses (PRISMA) flow diagram of literature search process and result.

In accordance with the PRISMA statement, the flowchart summarizing the literature search process is shown in Fig. 1. The initial search identified 636 potential studies, of which 507 were available after removing duplicates. A total of 487 articles were excluded after screening based on the title and abstract for eligibility, and another five articles were removed after full-text assessment. Finally, 15 articles met the inclusion criteria.

### Study and participant characteristics

Detailed characteristics of the included studies and the participants are summarized in Table 1. All were English studies except for one in Chinese^16^ and one in French^17^. All of the included studies were published from 1999 to 2017 except for one published in 1986, and they included a total of 1502 randomized participants (935 boys and 469 girls, three studies did not report the gender). The majority of the included participants were healthy children diagnosed with monosymptomatic nocturnal enuresis with or without previous treatment. Six out of 15 studies did not mention the status of previous treatment status. The other 9 studies excluded those who underwent treatment within 3 months of enrollment except that Evans *et al*. included children who underwent treatment with duration less than 4 weeks. Longstaffe *et al*. excluded those who were already treated with desmopressin or alarm. None of the studies used concomitant medications along with alarm or desmopressin therapy. The definition of enuresis varied, with most of the studies using three or more nights per week. Variable alarm devices were used, but most of the designs are classified as body sensor plus alarm. Six studies did not report the type of alarm. Four studies used an intranasal spray^7,17–19^, six used oral tablets^16,20–24^, four used oral melt^25–28^, and one used a nasal spray or oral tablets^29^. Dosage titration to 0.4 mg for oral tablets^20–24^ and 240 μg for oral melt^25,28^ was performed in seven studies under differed circumstances and time intervals from the initial treatment. Five studies performed structured withdrawals for desmopressin therapy^17,21,22,24,28^. Nine of the 15 studies did not report how the authors instructed the parents and children in the use of the bedwetting alarm, however all studies reported that the children were instructed to take the medication. All of the studies assessed response rates at the end of the medical treatment except for the study by Ma *et al*. which assess response rates 2 weeks after discontinuation of medication. The duration of therapy and follow-up period after discontinuation of therapy were 3–7 and 2–18 months, respectively.

**Table 1:** Characteristics of the 15 eligible randomized controlled trials.

Author, year	Country	Definition of enuresis	Inclusion criteria	Exclusion criteria	Age range	Male/Female	Duration of therapy (mo)	Duration of follow-up (mo)	Group size (A/D)	Enuresis alarm used	Alarm protocols and behavioral intervention	Desmopressin
Wille, 1986^13^	Sweden	3 nights or more per week during observation period	Healthy children not dry for 6 months or more after age 3	Previous treatment in the past 12 months	>6	N/A	3	2	25/25	3 Types (1) Eastleigh Al or AlP (2) N H Eastwood and Son Ltd, London (3) Sesam Dry-Bed, Astric Products Ltd, Brighton	N/A	Intranasal spray, 20 μg, Ferring, Malmo, Sweden
Faraj, 1999^12^	France	N/A	PMNE in healthy children	Previous treatment using desmopressin or alarm	6–16	93/42	3	3	73/62	Wet-stop ®, Laboratory Sega	N/A	Intranasal spray, 20 μg, Ferring SA. Structural withdrawal
Longstaffe, 2000^14^	Canada	12 nights or more during a 4-week period	MNE in healthy children with normal urinalysis and bladder capacity > 50% expected	Already on desmopressin or alarm treatment	>7	93/28	6	6	61/60	Details not provided	N/A	Intranasal spray (no further details provided)
Ng, 2004^16^	Hong Kong	3 nights or more per week during a 2-week observation	PNE in healthy Chinese children in Hong Kong	Previous treatment using desmopressin, alarm or tricyclic antidepressant	7–15	48/25	3	3	35/38	Wet-stop (Palco Laboratories, Santa Cruz, USA)	Instruct the parents and children to use the alarm	Oral tablet 0.2 mg initially and increased to 0.4 mg after 2 weeks or at any time thereafter if still more than 1 wet night per week
Ma, 2007^11^	China	1 night or more per month for at least 3 months	Those met ICD-10 criteria	No specification of previous treatment status	5–16	48/50	4	3	52/46	Details not provided	- Instruct the parents and children to use the alarm - Star chart reward system	Oral tablet 0.1 mg
Tuygun, 2007^25^	Turkey	3 nights or more per week for at least 3 months	MNE in healthy children	No specification of previous treatment status	6–13	50/34	3	3	35/49	Enurin (Aymed Analytic Medical Systems, Ankara, Turkey)	N/A	Intranasal spray (20–40 μg/day) or tablets (0.2–0.4 mg/day)
Vogt, 2010^17^	Germany	N/A	MNE in healthy children	Previous treatment in the past 12 months	5–15	Details not provided	3	6	19/24	Enutrain® (PROCON, Hamburg, Germany)	N/A	Oral tablet 0.2 mg for 2 weeks, and 0.4 mg the next 10 weeks. Structural withdrawal
Kwak, 2010^18^	Korea	N/A	MNE in healthy children	Previous treatment using desmopressin or alarm within the preceding 3 months	6–15	79/25	3	3	50/54	Malem Medical, Nottingham, UK	Instruct the parents and children to use the alarm	Oral tablet 0.2 mg initially and increased to 0.4 mg if <90% decrease after 2-week treatment. Structural withdrawal
Evans, 2011^19^	UK	6 nights or more over 2 weeks of screening	Untreated PNE, or who had been treated > 1 year ago and/or for <4 weeks	Treatment in the past 12 months and/or >4 weeks	5–16	182/69	6 (A), 3 or 6 if still wet (D)	12	59/192	Details not provided	N/A	Oral tablet 0.2 mg for 2 weeks. Kept 0.2 mg if 1 or less wet night afterwards, otherwise increased to 0.4 mg
Ahmed, 2013^21^	Saudi Arabia	2 nights or more per week over 3 consecutive months	Those met DSM-IV criteria	Previous treatment with desmopressin or alarm	6–14	95/41	3	3	45/46	Details not provided	Instruct the parents and children to use the alarm	Oral melt 120 μg but increased to 240 μg if still >1 wet night/wk after 2-week treatment
Tuncel, 2014^22^	Turkey	N/A	MNE in healthy children	No specification of previous treatment status	N/A	59/45	3	N/A	49/55	Enurin, Aymed Medical Inc.	N/A	Oral melt, 120 μg, Ferring
Bolla, 2014^23^	Italy	N/A	MNE in healthy children	No specification of previous treatment status	5–13	31/29	3 (or 6 if failed)	16 (A), 18 (D)	30/30	N/A	N/A	Oral melt, 120 μg
Önol, 2015^24^	Turkey	6 nights or more every 2 weeks	PMNE in healthy children	History of any treatment for PMNE within the preceding 3 months	6–15	80/38 (dropouts excluded)	6	6	45/73 (dropouts excluded)	Enurin, Aymed Medi-cal Inc.	Instruct the parents and children to use the alarm	Oral melt, 120 μg, and increased to 240 μg if no substantial response. Structural withdrawal.
Kasaeeyan, 2015^15^	Iran	2 times or more per week over 3 consecutive months	PNE in healthy children	No specification of previous treatment status	5–12	77/43	6	6	60/60	Details not provided	N/A	Intranasal spray, 1 spray (0.1 mg/mL) in each nostril one time
Fagundes, 2017^20^	Brazil	N/A	MNE in healthy children	No specification of previous treatment status	6–16	Details not provided	4–7 (A) and/or till full response, 4 (D)	12	30/20	2 Types (1) Wet-Stop 3®, PottyMD, Knoxville, TN (2) Bbell and carpet produced by the University of São Paulo Behavior Therapy Laboratory of the Psychology Institute	1.Patient/family training session on the use of the equipment 2. Superlearning 3. Overlearning	Oral tablet 0.2 mg and increased to 0.4 mg if <50% decrease after 30-day treatment. Structural withdrawal

MNE = Monosymptomatic nocturnal enuresis; PNE = Primary nocturnal enuresis; PMNE = Primary monosymptomatic nocturnal enuresis; A = Alarm; D = Desmopressin.

### Risk of bias assessment

**Figure 2:**
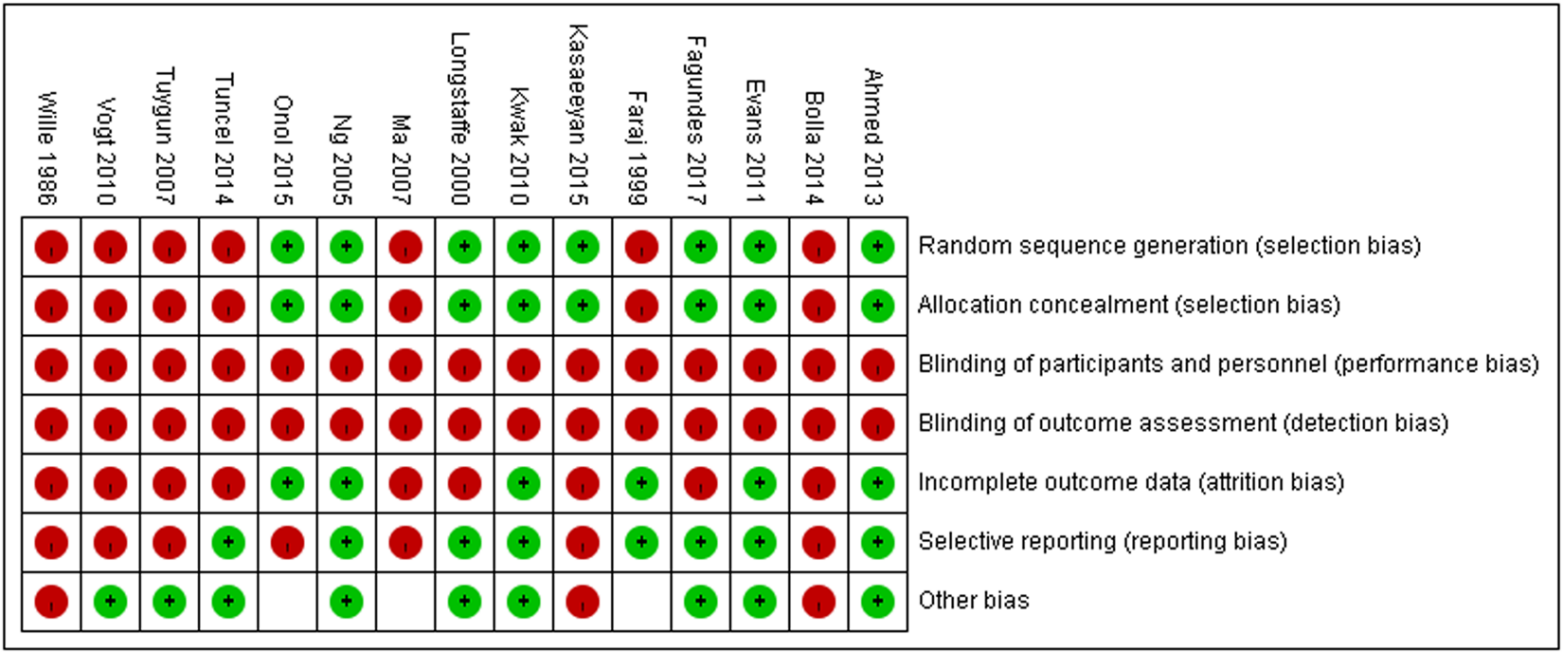
The summary of assessment of risk of bias in the included studies.

The summary of risk of bias within studies is presented in Fig. 2. None of the studies were blinded, and all of the studies were classified as high risk in performance bias and detection bias. An inadequate description of randomization and allocation concealment, and failure to report attrition were rated as a high risk of bias. Approximately half of the included studies described the randomization methods and analyzed attrition, and were classified as low risk in selection bias and attrition bias, respectively.

### Primary outcomes

#### Overall results

**Figure 3:**
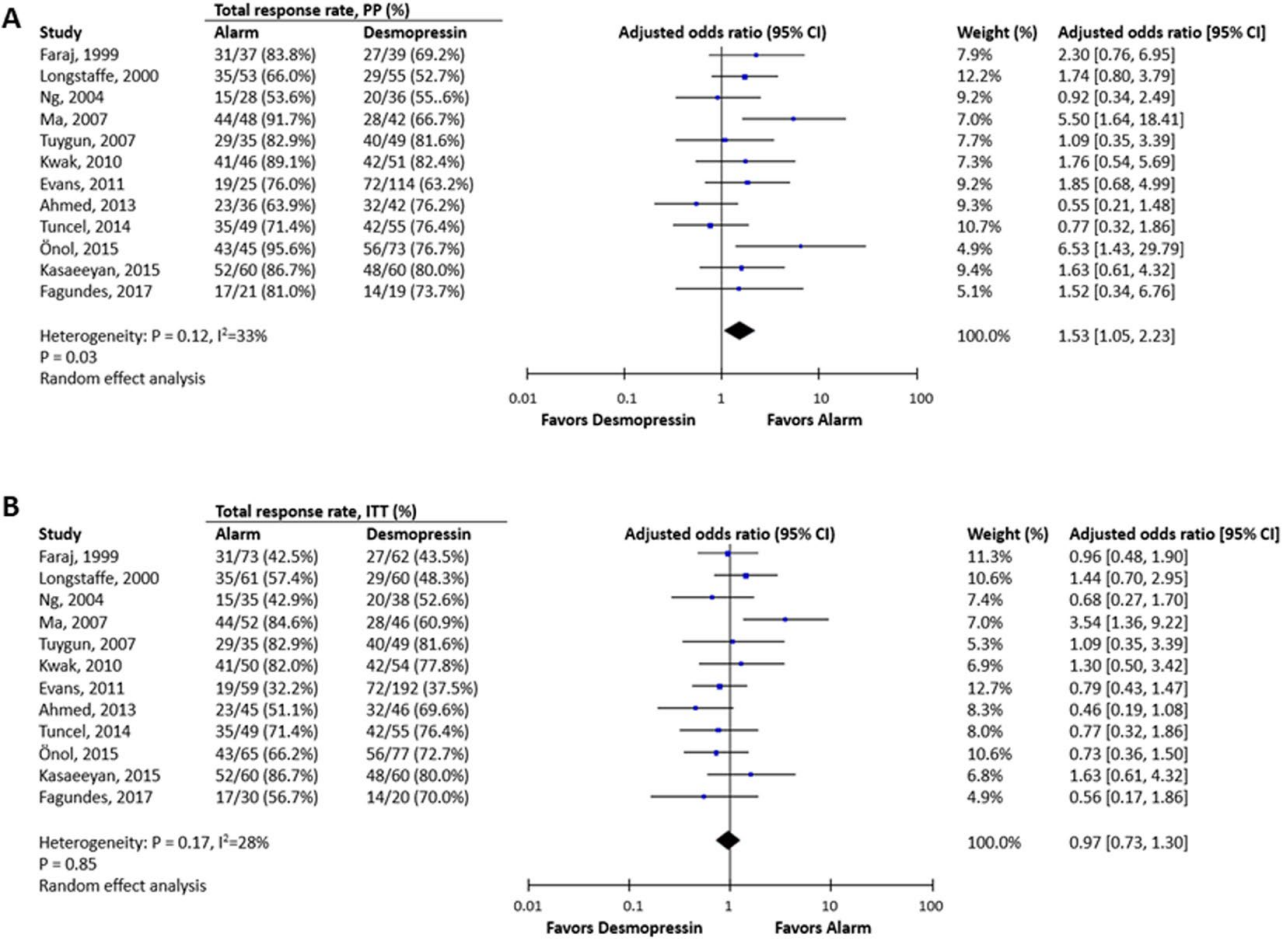
The comparison of efficacy between desmopressin and alarm in achieving at least partial response in per-protocol (PP) analysis (**A**) and in intention-to-treat (ITT) analysis (**B**).

The results of all pooled results of outcome parameters with subgroup analyses are shown in Table 2, and comparisons of baseline and post-treatment wet nights are listed in Table 3. Alarm therapy significantly outperformed desmopressin therapy in achieving at least a partial response in PP analysis (OR: 1.53, 95% CI 1.05 to 2.23, P = 0.03; I^2^ = 33%, Fig. 3A) but not in ITT analysis (OR: 0.97, 95% CI 0.73 to 1.30, P = 0.85; I^2^ = 28%, Fig. 3B). In both PP and ITT analyses, there were no significant differences between alarm therapy and desmopressin in full response rate and response rate, while desmopressin therapy had a significantly higher partial response rate in ITT analysis (OR: 0.63, 95% CI 0.42 to 0.93, P = 0.02; I^2^ = 22%).

**Table 2:** Meta-analysis and subgroup analysis of alarm and desmopressin, using different outcome measurements.

	No. of studies	Pooled OR Estimate (95% CI)	I^2^ estimate
Full response rate (ITT)	3	1.43 (0.49 to 4.12)	77%
Full response rate (PP)	3	1.72 (0.70 to 4.22)	65%
Response rate (ITT)	9	1.16 (0.8 to 1.66)	28%
Response rate (PP)	9	1.42 (0.92 to 2.20)	44%
Partial response rate (ITT)	9	0.63 (0.42 to 0.93)*	22%
Partial response rate (PP)	9	0.75 (0.49 to 1.14)	28%
≥50% response rate (ITT)	12	0.97 (0.73 to 1.30)	28%
*Variable formulations*			
Intranasal spray	3	1.24 (0.8 to 1.93)	0%
Oral tablet	5	1.07 (0.58 to 1.97)	56%
Oral melt	3	0.65 (0.41 to 1.03)	0%
*Dose titration*			
With titration	7	0.75 (0.54 to 1.03)	0%
Without titration	5	1.37 (0.85 to 2.20)	39%
≥50% response rate (PP)	12	1.53 (1.05 to 2.23)*	33%
*Variable formulations*			
Intranasal spray	3	1.82 (1.07 to 3.10)*	0%
Oral tablet	5	1.83 (1.03 to 3.27)*	21%
Oral melt	3	1.23 (0.35 to 4.30)	75%
*Dose titration*			
With titration	7	1.35 (0.80 to 2.27)	31%
Without titration	5	1.79 (1.01 to 3.18)*	43%
Relapse rate			
*Total*	10	0.25 (0.12 to 0.50)*	49%
Strict definition	3	0.12 (0.04 to 0.42)*	0%
Broad definition	7	0.31 (0.13 to 0.72)*	62%
*Withdrawal method*			
Structural withdrawal	3	0.36 (0.05 to 2.79)	60%
Abrupt withdrawal	7	0.22 (0.10 to 0.49)*	53%
*Variable formulations*			
Intranasal spray	2	0.21 (0.05 to 0.94)*	49%
Oral tablet	4	0.22 (0.06 to 0.78)*	64%
Oral melt	3	0.36 (0.03 to 5.09)	62%
Sustained response rate	4	2.89 (1.38 to 6.04)*	28%
Dropout rate	15	2.20 (1.41 to 3.42)*	28%
*Instructions for alarm*			
Provided	6	3.10 (1.47 to 6.52)*	35%
Not provided	9	1.75 (1.17 to 2.64)*	0%
*Variable formulations*			
Intranasal spray	4	1.72 (0.97 to 3.07)^†^	0%
Oral tablet	6	1.82 (0.89 to 3.73)	32%
Oral melt	4	4.77 (1.58 to 14.42)*	41%

Pooled OR > 1: favors alarm; Pooled OR < 1: favors desmopressin; *p < 0.05; ^†^p = 0.06.

**Table 3:** Comparison of number of wet nights before and after treatment.

Author, year	Mean wet night/wk (SD), pretreatment		Mean wet night/wk (SD), after treatment	
	Alarm	Desmopressin	Alarm	Desmopressin
Faraj, 1999	6.1	5.9	1.4	2.6
Ng, 2004	5.1 (1.5)	5.4 (1.4)	2.8 (2.2)	2.6 (2.4)
Tuygun, 2007	5.8 (1.6)	5.9 (1.6)	0.9 (1.9)	2.7 (2.7)
Kwak, 2010	5.4 (1.2)	5.8 (1.0)	N/A	N/A
Evans, 2011	5.5	5.6	N/A	N/A
Ahmed, 2013	4.6 (1.7)	5.0 (1.6)	2.1 (1.3)	1.8 (1.6)

The definitions of relapse in the included studies varied, and only three studies followed the definition suggested by the ICCS in 2006^15^. The pooled results revealed that desmopressin therapy had a significantly higher relapse rate than alarm therapy despite the varying definitions. Only four of the 15 studies reported a sustained response rate after discontinuation of treatment for more than 3 months, and the results showed that alarm therapy had a significantly better sustained response rate during follow-up (OR: 2.89, 95% CI 1.38 to 6.0; I2 = 28%).

#### Subgroup analyses

We performed subgroup analysis comparing desmopressin therapy with various formulations, with or without dose titration and structured withdrawal with alarm therapy regarding all outcome variables (Table 2). Among three administration routes, only desmopressin melt had a higher odds of achieving at least a partial response in ITT analysis, although the result was not statistically significant (pooled OR: 0.65, 95% CI 0.41 to 1.03, P = 0.07; I^2^ = 0%). Dose titration of desmopressin may have offered a better response, but again the result was not statistically significant (OR: 0.75, 95% CI 0.54 to 1.03, P = 0.08; I^2^ = 0%). There was no significant difference in relapse rate between alarm therapy and desmopressin of variable formulations and structured withdrawal.

#### Dropout rate

Overall, alarm therapy had a significantly higher odds of dropout than desmopressin therapy (OR: 2.20, 95% CI 3.41 to 4.29; I^2^ = 28%). Subgroup analysis (Table 2) revealed that the dropout rate was higher in the alarm group regardless of whether the authors reported that the parents/families were instruction on how to use the alarm. The subgroup analysis also revealed that alarm therapy had the highest odds of dropout when compared with the oral melt form of desmopressin (OR: 4.77, 95% CI 1.58 to 14.4).

#### Analysis of reasons for dropouts and adverse events

Table 4 summarizes the detailed reasons for dropouts and adverse events in each study. Eight of the 15 included studies provided related information. “Lack of efficacy”, “discomfort”, and “disturbing other family members” were the three most common reasons for patients to withdraw from alarm therapy. For desmopressin therapy, most studies reported a low dropout rate (<10%), with “loss of follow-up” and “fear of drug dependence” listed as the top reasons. The dropout rate from alarm therapy was as high as 57.6%^23^.

**Table 4:** Analysis of dropouts and adverse events.

Author, year	Dropout causes		Adverse events	
	A	D	A	D
Wille, 1986	1 (4%) Withdrew 1 (4%) UTI 1 (4%) Became dry before started treatment	1 (4%): Unspecified:	21 (84%) False alarms 5 (20%) Alarm didn’t work 15 (60%) Alarm didn’t awake the child 15 (60%) Other family members woke instead	5 (20%) nasal discomfort, 1 (4%) occasional nose bleed, 2 (8%) bad taste in the throat
Faraj, 1999	36 (49.3%) Did not purchase the alarm	23 (37.1%) Non-compliant or lost to follow-up	Not mentioned	Not mentioned
Ng, 2004	5 (14.3%) Ineffective 3 (8.6%) Disturb other family members 2 (5.7%) Discomfort 1 (2.9%) Too cold to get up	2 (5.3%) Fear of drug dependency	None	None
Kwak, 2010	1 (2.0%) Poor compliance 3 (6.0%) Withdrew consent			
	1 (1.9%) Abdominal pain and voiding difficulty 1 (1.9%) Loss of follow-up 1 (1.9%) Withdrew consent	None	1 (1.9%) Abdominal pain and voiding difficulty	
Evans, 2011	1 (1.7%) Due to TEAEs* 8 (13.6%) Lack of efficacy 13 (22.0%) Patient preference 12 (20.3%) Other reasons	10 (5.2%) Due to TEAEs* 28 (14.6%) Lack of efficacy 19 (9.9%) Patient preference 21 (10.9%) Other reasons *During follow-up* 2 (1.0%) due to lack of efficacy 5 (3.0%) other reasons	*During treatment* 8 (14.0%) TEAEs*, including 1 (1.7%) Severe: Anxiety, probably related *During follow-up* 1 (4%) Unrelated	*During treatment* 58 (30.2%) TEAEs*, including 3 (1.6%) Headache, related 3 (1.6%) Severe cases: 1 (0.5%) Dysuria and micturition urgency, possibly related to medication 1 (0.5%) Appendicitis, unlikely 1 (0.5%) Rash, unrelated *During follow-up* 1 (0.9%) mild and related 3 (2.6%) unrelated
Ahmed, 2013	1 (2.2%) Loss of follow-up 3 (6.7%) Discomfort 4 (8.9%) Ineffective 1 (2.2%) Disturb other family members	1 (2.2%) Loss of follow-up 3 (6.5%) Fear of drug dependence	None	None
Bolla, 2014	N/A	N/A	Not mentioned	4 (13.3%) Temporary asymptomatic hyponatremia
Önol, 2015	8 (12.3%) Discomfort with device 5 (7.7%) Lack of efficacy 7 (10.8%) Loss follow-up	4 (5.2%) Loss of follow-up	Not mentioned	Not mentioned

*Treatment-emergent adverse events (TEAEs) were defined as any unfavorable and unintended sign, symptom or disease temporally (not necessarily causally) associated with use of the product.

Advert effects were uncommon with both therapies. Only Wille *et al*.^18^ mentioned a high percentage of false alarms (84%) and waking other family members instead of the child (60%). For desmopressin therapy, Wille *et al*. reported nasal discomfort (20%), occasional nose bleeds (4%) and a bad taste in the throat (8%) with the intranasal administration of desmopressin. Among all of the included studies, only four cases of temporary asymptomatic hyponatremia (13.3%) resulting from desmopressin melt were reported^27^. In general, alarm and desmopressin therapy were reported as being well-tolerated with a good safety profile.

#### Publication bias

**Figure 4:**
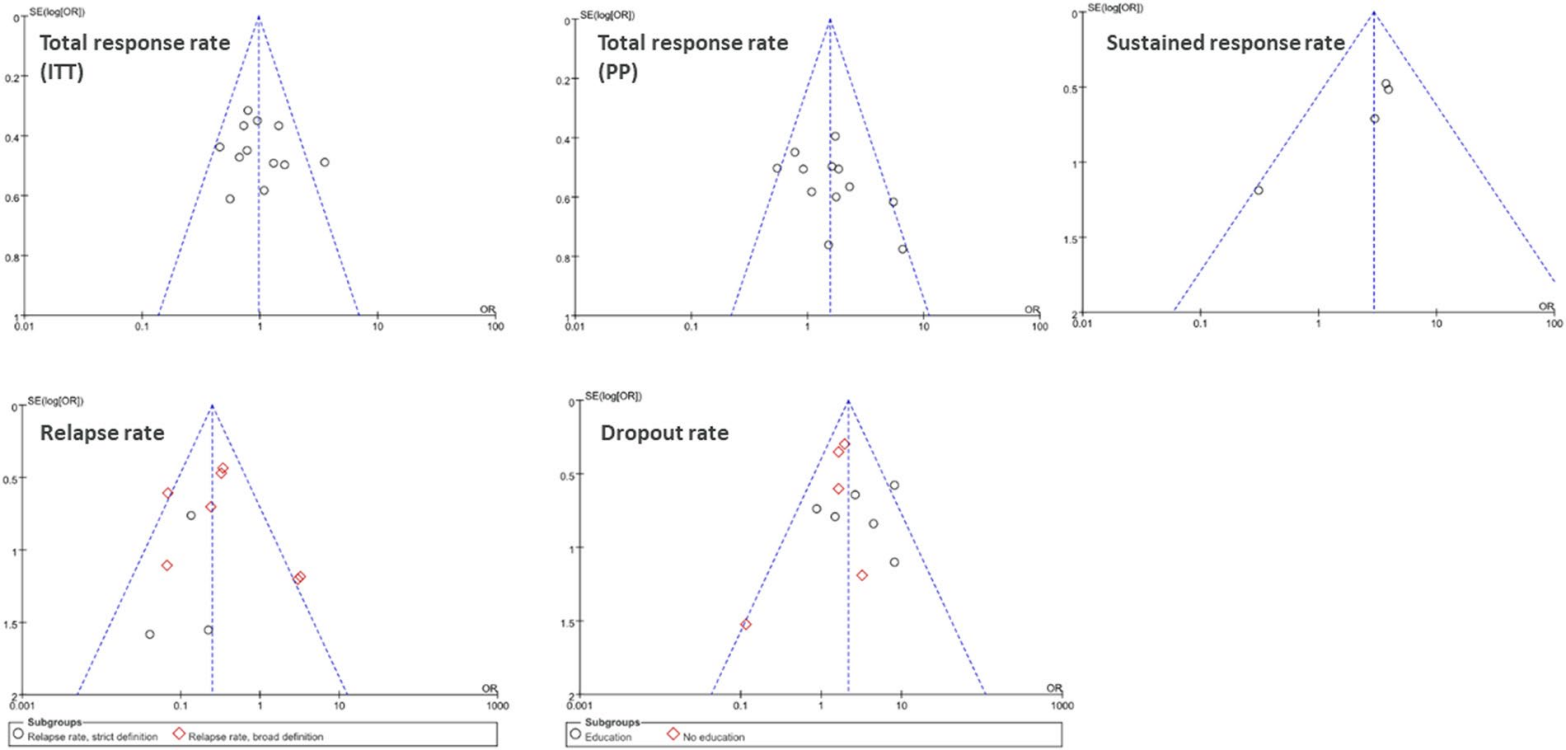
Low probability of publication bias is shown in funnel plots of total response rate (ITT, **A**), total response rate (PP, **B**), sustained response rate (**C**), relapse rate (**D**), and dropout rate (**E**).

Possible publication bias was analyzed by funnel plots using all evaluated outcomes and results. There are totally five representative plots, including total response rate (ITT), total response rate (PP), relapse rate, sustained response rate and dropout rate, shown in Fig. 4. They all demonstrated low probability of publication bias.

## Discussion

This up-to-date detailed systemic review and meta-analysis is the first to provide information in English on the efficacy and adverse effects of alarm therapy with desmopressin therapy for monosymptomatic nocturnal enuresis in children. The results demonstrated that alarm therapy had higher odds of achieving a >50% reduction in wet nights in PP analysis, and a lower relapse rate and better sustained response rate. However, alarm therapy did not outperform desmopressin therapy in ITT analysis due to a significantly higher dropout rate. Subgroup analyses revealed that strategies with dose titration and melt form desmopressin did not significantly differ from alarm therapy in achieving a >50% reduction in wet nights in ITT analysis. In general, both alarm and desmopressin therapy for pediatric nocturnal enuresis were well tolerated.

The alarm requires the cooperation of family members for a prolonged period, and this probably explains why children using an alarm have been reported to have a 30–50% dropout rate^17,23,28,30,31^. Only 6 out of the 15 included studies reported that parents and children were trained to use the instruments and only two studies reported their methods of behavioral intervention (Table 1). Improper introduction/training and a lack of clear protocol with regards to the use of the alarm may hamper compliance. Familiarity, confidence and adequate training of physicians and study nurses are important for a successful outcome and better compliance^9^. However, the overall dropout rate was lower in our meta-analysis (alarm: 19.5%, 134/688; desmopressin: 15.4%, 129/838). The lack of efficacy, patient preference and other reasons were the top reasons for dropping out (57.6%, 34/59) in Evans *et al*.’s^23^ study. The reasons for withdrawal, including discomfort, lack of efficacy and loss of follow-up, resulted in 30.8% of the dropouts (20/65) in Önol *et al*.’s study^28^. The efficacy of such alarms is hampered by the high dropout rate and, therefore, the overall proportion of patient who had a >50% reduction in wet nights were comparable between the alarm and desmopressin groups in ITT analysis (OR: 0.97, 95% CI 0.73 to 1.30). The enuresis alarms outperformed desmopressin in achieving a sustained response (alarm: 67.8%, 78/115; desmopressin: 45.5%, 60/132; P = 0.005) and lower relapse rate (alarm: 12.9%, 37/287; desmopressin: 37.6%, 118/314; P = 0.0001) which are in line with the results of the previous studies. The results suggested that alarm could had a significant long-term treatment success rate than desmopressin.

In our subgroup analyses of the three different formulations of desmopressin, oral melt demonstrated the lowest dropout rate (oral melt: 3.8%, 8/208; oral tablet: 24.6%, 92/374; intranasal spray: 14.0%, 29/207). In addition, the alarm group had the highest odds (OR: 4.77, 95% CI 1.58–14.42) of dropout when compared with the oral melt form, and a lower odds of achieving a >50% reduction in wet nights (OR: 0.65, 95% CI 0.41–1.03) when compared with the oral melt form in ITT analysis, although without statistical significance. Moreover, the relapse rate was lower with the oral melt form (oral melt: 18.8%, 12/64; oral tablet: 40.2%, 47/117; intranasal spray: 38.1%, 32/84). A previous meta-analysis of desmopressin withdrawal strategies including four randomized controlled trials with a total of 500 subjects indicated that structured withdrawal offered a better relapse-free rate^32^. In the review, the relapse rate was also lower with a structured withdrawal (27.5%, 14/51) than abrupt withdrawal (41.1%, 104/253). However, there is no evidence to support whether structured withdrawal of desmopressin or alarm is better with regard to relapse rate.

The adverse effects of alarm are minimal except malfunction, skin rash, anxiety and disturbing the family. Possible side effects of desmopressin included muscle weakness, spasms, hyponatremia, gastrointestinal symptoms, seizures and other neurological symptoms^33^. FDA remove the indication of intranasal desmopressin on the management of nocturnal enuresis in children aged ≥6 years as there were 61 cases of hyponatremia-related seizures and 2 of them resulted in death^34^. Despite the fact that there existed low reported morbidity rates of desmopressin in the included studies and less hyponatremia/seizures were associated with tablet formulation, the possible side effects should be kept in mind.

The cost of treatment and insurance payment system may influence the preference to a specific therapy. Desmopressin therapy is more expensive than an enuresis alarm^35^. In the UK, 16 weeks of drug treatment (the usual time allowed for fourteen consecutive dry nights to be attained using an alarm) costs £116 for desmopressin tablets (200 μg), and enuresis alarms (including batteries & sensor) typically cost £33.60. In addition, the costs associated with those needing the continued use of desmopressin, prolonged use due to structured drug withdrawal strategy, and medications needed to treat relapse were not taken into account^35^. Therefore, the costs associated with desmopressin are much higher than reported. In the included studies, all studies did not mention the financial support from the pharmaceutical company. Because all the included studies were randomized controlled trials, participants might not pay for the fee of medication and higher costs of desmopressin, therefore, did not lead to dropout of participants.

There are several limitations to this study. First, the severity of enuresis in the enrolled patients, types of medication, administrative routes, doses, titration and withdrawal strategies, behavioral strategies, patient education and types of alarm varied from study to study leading to heterogeneity in the analyzed parameters. Therefore, the pooled analysis for comparisons used a random effects model. Second, the variables or ambiguous definitions of outcome parameters used in each study resulted in difficulty in unifying and comparing the treatment results between studies. To overcome this limitation, we tried to analyze the results following the standardized terminology proposed by the ICCS^6,36^. None of the studies reported how many enuretic children in the desmopressin group required the continued use of desmopressin. Lastly, the risk of bias was high in many of the included studies regarding unspecified randomization schemes and dropouts. The strength of this study is that we collected 15 randomized controlled trials, published in English, French and Chinese, from 12 different countries over three decades.

With regards to the management of children with monosymptomatic enuresis, the results of this study revealed that alarm and desmopressin therapy were comparable in efficacy with regards to achieving a >50% reduction in baseline wet nights in the intention to treat analysis. However, enuresis alarms offered a superior sustained treatment response and a lower relapse rate than desmopressin, especially in compliant children and parents.

## Methods

### Search strategy and eligibility criteria

Two independent investigators (CCP and SJC) conducted a systematic search of Medline, Embase, Science Direct, Google Scholar, Cochrane Central Register of Controlled Trials (CENTRAL), Wiley, and ClinicalTrial.gov from the earliest record to April 2017 with no restrictions on language using the keywords: alarm and desmopressin and enuresis. The reference lists of the included articles and review articles were manually reviewed, and external peer reviewers were asked to identify any additional trials. Only randomized controlled trials (RCTs) comparing the efficacy between alarm and desmopressin therapy in pediatric patients with monosymptomatic nocturnal enuresis were eligible for inclusion.

### Data extraction

We followed the Preferred Reporting Items for Systematic Reviews and Meta-analyses (PRISMA) guidelines with the associated flow chart to report the numbers of included and excluded studies at each stage. Two investigators (CCP and SJC) independently reviewed the citations and abstracts for relevance and the eligibility criteria. The full text of articles was assessed if the eligibility was not clear from the abstract. The authors of the studies were contacted by email to ask for incomplete data or to answer queries in cases of incomplete data.

One investigator abstracted details of the case definitions for monosymptomatic nocturnal enuresis, patient population, details of interventions, definitions of various outcome measurements, duration of follow-up, number and nature of dropouts and adverse events, and the other investigators checked for accuracy.

### Risk of bias assessment

The Cochrane Collaboration risk of bias tool was used to rate the risk of bias as low, unclear, or high. Discrepancies were resolved through consensus and any further disagreement was reviewed by a senior physician researcher. A funnel plot with Egger regression test was generated to determine potential publication bias.

### Definition of outcomes

The outcomes of the study were treatment response, relapse rate, sustained response rate and dropout rate. To ameliorate discrepancies, we used the definition of treatment outcomes suggested by the ICCS in 2006^15^ as follows: nonresponse was defined as a 0–49% decrease, partial response as a 50–89% decrease, response as a ≥90% decrease, and full response as a 100% decrease or less than one occurrence of symptoms per month.

### Data synthesis and analysis

Data analyses were performed using RevMan, version 5.3.5 (www.cochrane.org). We conducted pooled analysis of dichotomous outcomes using pooled odds ratios (ORs) and 95% confidence intervals (95% CIs) with a random model due to heterogeneity in the enrolled studies. Both intention-to-treat (ITT) and per-protocol (PP) analyses were performed to evaluate treatment outcomes due to the large percentage of dropouts in the alarm group. We stratified the analyses according to the route of desmopressin administration, dose titration of desmopressin and withdrawal method of desmopressin in particular outcome measurements. The I^2^ statistic served as a measure of study heterogeneity.

## Electronic supplementary material

PRISMA checklist

## Data Availability

All data generated or analyzed during this study are included in this published article.
